# Non-linear Entropy Analysis in EEG to Predict Treatment Response to Repetitive Transcranial Magnetic Stimulation in Depression

**DOI:** 10.3389/fphar.2018.01188

**Published:** 2018-10-30

**Authors:** Reza Shalbaf, Colleen Brenner, Christopher Pang, Daniel M. Blumberger, Jonathan Downar, Zafiris J. Daskalakis, Joseph Tham, Raymond W. Lam, Faranak Farzan, Fidel Vila-Rodriguez

**Affiliations:** ^1^Non-Invasive Neurostimulation Therapies (NINET) Laboratory, Department of Psychiatry, University of British Columbia, Vancouver, BC, Canada; ^2^Department of Psychology, Loma Linda University, Loma Linda, CA, United States; ^3^Temerty Centre for Therapeutic Brain Intervention and Campbell Family Research Institute, Centre for Addiction and Mental Health, Toronto, ON, Canada; ^4^Department of Psychiatry, University of Toronto, Toronto, ON, Canada; ^5^MRI-Guided rTMS Clinic and Krembil Research Institute, University Health Network, Toronto, ON, Canada; ^6^Department of Psychiatry, University of British Columbia, Vancouver, BC, Canada; ^7^School of Mechatronic Systems Engineering, Simon Fraser University, Surrey, BC, Canada

**Keywords:** EEG, rTMS, major depressive disorder, permutation entropy, empirical mode decomposition, biomarker

## Abstract

**Background:** Biomarkers that predict clinical outcomes in depression are essential for increasing the precision of treatments and clinical outcomes. The electroencephalogram (EEG) is a non-invasive neurophysiological test that has promise as a biomarker sensitive to treatment effects. The aim of our study was to investigate a novel non-linear index of resting state EEG activity as a predictor of clinical outcome, and compare its predictive capacity to traditional frequency-based indices.

**Methods:** EEG was recorded from 62 patients with treatment resistant depression (TRD) and 25 healthy comparison (HC) subjects. TRD patients were treated with excitatory repetitive transcranial magnetic stimulation (rTMS) to the dorsolateral prefrontal cortex (DLPFC) for 4 to 6 weeks. EEG signals were first decomposed using the empirical mode decomposition (EMD) method into band-limited intrinsic mode functions (IMFs). Subsequently, Permutation Entropy (PE) was computed from the obtained second IMF to yield an index named PEIMF2. Receiver Operator Characteristic (ROC) curve analysis and ANOVA test were used to evaluate the efficiency of this index (PEIMF2) and were compared to frequency-band based methods.

**Results:** Responders (RP) to rTMS exhibited an increase in the PEIMF2 index compared to non-responders (NR) at F3, FCz and FC3 sites (*p* < 0.01). The area under the curve (AUC) for ROC analysis was 0.8 for PEIMF2 index for the FC3 electrode. The PEIMF2 index was superior to ordinary frequency band measures.

**Conclusion:** Our data show that the PEIMF2 index, yields superior outcome prediction performance compared to traditional frequency band indices. Our findings warrant further investigation of EEG-based biomarkers in depression; specifically entropy indices applied in band-limited EEG components. Registration in ClinicalTrials.Gov; identifiers NCT02800226 and NCT01887782.

## Introduction

Major depressive disorder (MDD) is a global public health concern (World Health Organization [WHO], 2016) as the disease is the leading cause of disability (Zarate et al., 2013) and affects approximately 5.4% of the population worldwide (Ferrari et al., 2013). Furthermore, it is estimated that 40% of those living with the disorder do not respond to first line treatments such as pharmacological or psychosocial treatments and have treatment resistant depression (TRD) (Berlim et al., 2008).

Repetitive transcranial magnetic stimulation (rTMS) is a safe and effective treatment for TRD with 50–55% response and 30–35% remission rates (Galletly et al., 2011; McDonald et al., 2011), and rTMS is considered a first-line treatment option for TRD (Milev et al., 2016). rTMS induces an electric field in the brain strong enough to depolarize neurons and trigger action potentials, and the treatment is delivered non-invasively by applying a coil in contact with the scalp. Neuroimaging studies have shown a degree of hypoactivity in the left dorsolateral cortex (L-DLPFC) in MDD (Hallett, 2007), and therefore rTMS protocols that increase cortical excitability have been applied to the L-DLPFC for the treatment of MDD.

The prescription of rTMS, similar to antidepressant medication prescription, is currently based on clinical assessment and a process of trial and error. Identification of effective biomarkers that can inform clinical decisions is lacking, and this absence may contribute to higher health-care costs (Silverstein et al., 2015). Developing reliable biomarkers may have profound implications for clinical practice as it would shift the prescription process to a more precise and personalized approach that would further improve clinical outcomes and efficiency during treatment initiation (Collins et al., 2011).

The search for biomarkers of response has expanded to molecular, neurophysiological and neuroimaging methods (Silverstein et al., 2015). The resting-state electroencephalogram (rsEEG) has merited particular interest due to its ease of use, cost-effectiveness and non-invasive nature which are optimal characteristics for its implementation in clinical settings (Baskaran et al., 2012; Shalbaf et al., 2015b; Wade and Iosifescu, 2016).

Several frequency-based rsEEG measures have been proposed as predictors of response in TRD in the context of rTMS. Examples include theta (4–7 Hz) activity in the subgenual zone of the anterior cingulate cortex (Narushima et al., 2010), anterior alpha (8–12 Hz) peak frequency (Arns et al., 2012), prefrontal cordance (combination of absolute and relative EEG power at different bands), (Bares et al., 2015; Erguzel et al., 2015) and Lempel-Ziv analysis on the alpha band (Arns et al., 2014). However, these frequency-based methods are susceptible to artifacts and are more suitable for the analysis of stationary signals. Furthermore, these frequency-based measures require a Fourier transform, and this transform precludes precise estimation of temporal patterns in EEG (Huang et al., 1998).

Complexity and non-linear behavior are characteristics of typical brain functioning, (Elbert et al., 1994) and therefore the application of non-linear dynamics analyses to the EEG signal may prove to be a better measure of neural activity (Hosseini et al., 2010). Recently, a non-linear parameter called permutation entropy (PE) (Bandt and Pompe, 2002; Cao et al., 2004) has been developed to dissect the complexity of EEG signals by deciphering the local order structure of a dynamical time series (Cao et al., 2004). In addition, PE properly tracks the dynamics of brain activity (Shalbaf et al., 2013), is conceptually simple, computationally efficient, and robust against artifacts (Shalbaf et al., 2015a).

However, PE can be underestimated if the signals are superimposed with local or global trends. Research has suggested that properly removing the trends in biological signals with a decomposition approach may improve the performance of non-linear signal analysis (Lo et al., 2009; Tsai et al., 2012). Different variations of decompositions are suitable due to their ability to derive dynamical features from the signals with an enhanced resolution (Kevric and Subasi, 2017). One such method, empirical mode decomposition (EMD), was developed for analyzing non-stationary data (Huang et al., 1998). EMD can decompose a complicated signal without a basis function, such as sine or wavelet functions, into several intrinsic mode functions (IMFs) that are embedded in the original signal. The decomposition procedure is adaptive, data-driven and highly efficient (Kevric and Subasi, 2017). Therefore, entropy index applied in band-limited EEG component extracted with EMD method may optimally quantify non-linear neuronal oscillations.

The purpose of this study is to examine rsEEG features as predictors of treatment response in TRD patients receiving excitatory rTMS to the L-DLPFC. We hypothesized that rsEEG decomposition components will hold different energies for different patients and that these would differentiate responders (RP) from non-responders (NR). Furthermore, we hypothesized that non-linear methods would be better suited and more efficient predictors of rTMS treatment response compared to traditional linear frequency-band power metrics.

## Materials and Methods

### Participants and Experimental Procedures

The neurophysiology dataset was part of two randomized, single-blinded trials in which patients with TRD were assigned to receive either intermittent theta burst stimulation (iTBS) or high frequency left (HFL) rTMS protocols to the left DLPFC (parameters discussed below). Patients received a 4–6 week course of rTMS.

Participants were part of two separate clinical trials with identical inclusion and exclusion criteria registered in ClinicalTrials.Gov, identifier NCT02800226 and NCT01887782 (Blumberger et al., 2018). All participants provided informed consent and both experimental protocols were approved by both the UBC Clinical Research Ethics Board as well as the Vancouver Coastal Health Research Institute.

Subjects were 62 TRD patients and 25 healthy comparison (HC) subjects. Demographic characteristics were collected at baseline (Table 1). All participants completed rsEEG at baseline, prior to receiving treatment. Six patients did not complete the full 4 weeks of rTMS treatment and were excluded from analysis. Data from an additional 5 patients were removed from analysis due to random noise after quality control analysis. Thus, a dataset with a total of 76 participants was used in this study, including 51 TRD patients (26 randomized to iTBS and 25 to HFL) and 25 HC. The inclusion and exclusion criteria of patients and HC are outlined in Supplementary Material.

**Table 1:** Demographics and clinical characteristics of HC and TRD patients by responder and non-responder groups.

	TRD patients (*n* = 51)			
	Responders (*n* = 31)	Non-responders (*n* = 20)	Healthy volunteers (*n* = 25)	*p*
Sex (F/M)	19/12	12/8	18/7	ns^a^
Age (*SD*)	43.4 (11.6)	42.5 (14.1)	39.8 (13.1)	ns^b^
Years of education (*SD*)	15.2 (2.5)	15.0 (1.9)	16.0 (1.9)	ns^b^
Handedness (R/L/A)	26/5/0	15/3/2	21/2/2	ns^a^
HDRS (*SD*)	22.5 (4.3)	2249 (4.0)	-	ns^b^
Treatment (HFL/iTBS)	13/18	12/8	-	ns^a^

*M, male; F, female; SD, standard deviation; L, left; R, right; A, ambidextrous; HDRS, 17-item Hamilton Depression Rating Scale. ^*a*^Chi-square test. ^*b*^Two sample *t*-test to compare healthy controls to patients, since in the original analysis, a one-way ANOVA was used to compare age and education across the 3 groups*.

### Stimulation Technique and Parameters

Stimulation techniques have been previously described (Ge et al., 2017). Briefly, a MagPro X100 stimulator with a Cool-B70 fluid-cooled coil was used to deliver rTMS for all patients (Magventure, Farum, Denmark). Resting motor threshold was determined by visual inspection of right interpolicis brevis muscle contraction with the aid of the TMS Motor Threshold Assessment Tool (Dobek et al., 2016). All treatments were delivered at 120% resting motor threshold (Blumberger et al., 2018). Following randomization, TRD patients received either HFL stimulation or iTBS over the left DLPFC, using a Neuronavigation system (Visor 2.0, ANT Neuro, Enschede, Netherlands) and the target location specified by reverse coregistration from a stereotaxic coordinate on the standard Montreal neurological institute (MNI-152) template brain [x - 38, y + 44, z + 26] identified as optimal based on functional connectivity and clinical outcome (Dobek et al., 2016).

### Clinical Measures

Primary clinical outcome was measured using the 17-item Hamilton Depression Rating Scale (HDRS). For each patient, HDRS scores were collected at baseline and at the end of the rTMS course. Interviewers were blinded to patient treatment allocation. Responders (RP) were defined as those having a 50% or greater reduction in HDRS scores between baseline and end of treatment. Out of the 51 patients included in the analysis, there were 31 responders and 20 non-responders to rTMS treatment.

### Pre-treatment EEG Acquisition

rsEEG was collected using Brain Products EEG systems (Brain Products, Gilching, Germany) at two UBC sites part of the Canadian Biomarker Integration Network in Depression (Lam et al., 2016). The process of acquiring data with different systems has been carefully considered and addressed (Farzan et al., 2017a). Continuous rsEEG was recorded using 31 (site A) or 64 (site B) recording sites determined using the 10–20 system of electrode placement, an EasyCap electrode cap, and sintered Ag-AgCl electrodes. rsEEG data were recorded using a QuickAmp amplifier (Brain Products, Gilching, Germany; 1000 Hz A/D rate; 0.10 Hz high pass, 499 Hz low pass; common average reference; impedances ≤ 10 kΩ). rsEEG was obtain within 7 days of treatment initiation in all participants (mean 3.7 days).

Participants were given the same resting state instructions, “Please close your eyes for 3 min while we collect your brain activity at rest. Let your mind wander and try not to fall asleep.” All rsEEGs were conducted in a sound-attenuated room with reduced lighting to limit distraction and noise. Two sets of bipolar electrodes were placed around the participant’s eyes for collecting Electrooculogram (EOG) to track eye movements for artifact rejection.

### EEG Preprocessing

Two levels of pre-processing steps were implemented in order to standardize the EEG data collected from both sites. These steps are done in MATLAB (The Mathworks, Inc., Natick, MA, United States) via the open-source EEGLAB toolbox (Delorme and Makeig, 2004).

The aim of the first pre-processing step was to minimize raw data heterogeneity across two sites and prepare the data for integration. First, data from Site B were reduced because site B had more recording electrodes, and thus some electrodes were removed from analyses to match the same number of electrodes as Site A. The electrode locations in both caps were identical as per manufacturer description (Brain Products, Gilching, Germany in both site A and site B). Second, data from Site B were re-referenced to common average reference such that data from the two sites possess the equivalent electrode reference. Then, length of recording for all participants modified to have the same length. Also, separate frequency analysis and statistical tests from the same two healthy volunteers were done to show data were equivalent in quality across the two sites.

The second pre-processing step was to implement EEG artifact removal, since these artifacts interfere with the identification of true neurophysiological signal. First, the sampling rate of all data was decreased from 1000 to 256 samples per second to reduce white noise. Second, data segments contaminated with large-amplitude or random noise sources that cannot be extracted through filtering were removed with GUI workflow. Third, the high and low band pass filters were set to at 0.5 and 55 Hz respectively, to remove low and high frequency noise. The notch filter was also set at 60 Hz to remove industrial noise. Finally, blind source separation techniques via independent component analysis (ICA) (Makeig et al., 1996) were used to extract eye movements and blinks, muscle activity, and cardiac signals in order to separate neural activity from these sources of noise.

### Linear EEG Analyses

Time-frequency analyses using short time windowed Fast Fourier Transform (FFT) applied to resting EEG epochs were computed using MATLAB and EEGLAB software (Delorme and Makeig, 2004). Some conventional frequency band measures such as Delta (1–4 Hz), Theta (4–8 Hz), Alpha (8–12 Hz), Beta (12–24 Hz) and Gamma (30–50 Hz) relative powers were extracted from the rsEEG signals for both HC and TRD groups. 1024 points discrete FFT with a 100% Hanning window was computed over segments of 8 s with an overlap of 6 s and the average of all segments in a recording was considered the relative powers index for each participant.

### Non-linear EEG Analyses

Non-linear features were extracted from the eyes-closed resting state EEG signals of both HC and TRD groups. Details of the algorithm will be described in following sections.

#### Empirical Mode Decomposition (EMD)

EMD is a method of signal analysis (Huang et al., 1998) that has recently been applied to biological signals (Shalbaf et al., 2012). Using EMD, any complex signal can be decomposed into a small number of intrinsic mode functions (IMFs) through a sifting process. The IMFs should fulfill two requirements:

(1) The IMF is symmetric with respect to the local mean.
(2) Each IMF has the same number of zero crossings and extrema, or they differ at most by one.

**FIGURE 1:**
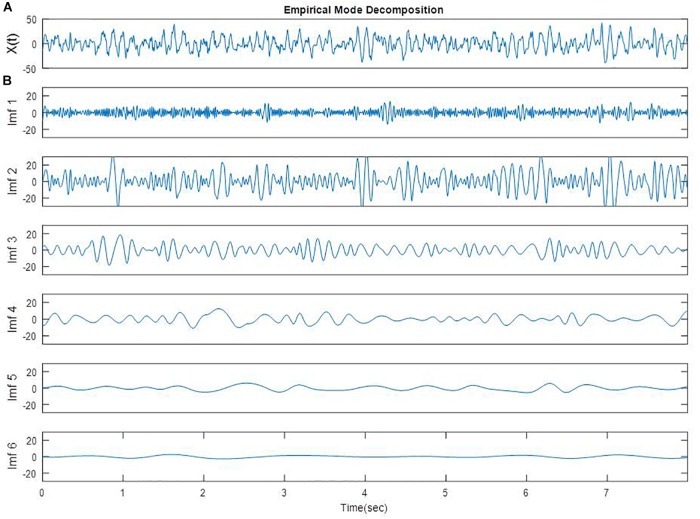
A segment of EEG signal from one participant in FC3 site **(A)** [X(t)] and EMD of the same segment (**B**, Imf 1 to Imf 6).

The detailed algorithm has been previously described and the number of IMFs was established to be six (Shalbaf et al., 2012). An EEG epoch of 8 s from one participant in FC3 electrode is plotted in Figure 1A. The EMD of this EEG epoch is composed of six IMFs which are shown in Figure 1B. These IMFs are almost orthogonal components.

#### Permutation Entropy (PE)

Permutation entropy (PE) is a new non-linear parameter that quantifies the amount of regularity in EEG data (Bandt and Pompe, 2002; Cao et al., 2004). This feature converts a given EEG series into a sequence of ordinal patterns, meaning that a non-stationary series can be transformed to an almost stationary ordinal series. The smallest and the largest values of PE are zero and one, with zero reflecting a highly regular time series and one reflecting equal probability of all permutations. The detailed algorithm and parameter sets have been previously published (Shalbaf et al., 2013).

#### Permutation Entropy Intrinsic Mode Functions (PEIMF2)

The EEG signals were first decomposed by applying the EMD method into symmetric and band-limited IMFs which are arranged from high to low frequency components. EMD decomposition was computed over segments of 8 s with an overlap of 6 s in order to consistently track the transient changes in the EEG recording. Then, PE was computed from each of the 8-s IMF2 segments. The average PE of all segments in a recording was considered the PEIMF2 index for each participant.

### Statistical Analysis

Differences in neurophysiological variables between RP, NR, and HC groups were examined using 1-way analysis of variance (ANOVA). The normality of the data was investigated before performing analyses, and a *p*-value of 0.01 was set as the criteria for statistical significance for greater stringency than conventional levels.

A Receiver Operator Characteristic (ROC) curve was plotted as a two-dimensional depiction of the classifier’s performance in predicting treatment outcomes using the proposed biomarkers. The two axes of this graph represent tradeoffs between errors (false positives) and successes (true positives) that a classifier makes between two classes. To corroborate the results of this analysis, the area under the ROC curve, abbreviated as AUC was calculated.

## Results

### Demographic and Clinical Characteristics

Table 1 summarizes the demographic and clinical characteristics of all participants. The responders, non-responders, and healthy comparison groups had similar age, sex, years of education, and handedness.

There were no differences between RP and NR as well as between MDD and HC in age and sex. Also Baseline HDRS scores of responders were similar to that of non-responders.

### Response Prediction Based on PEIMF2 Index

**FIGURE 2:**
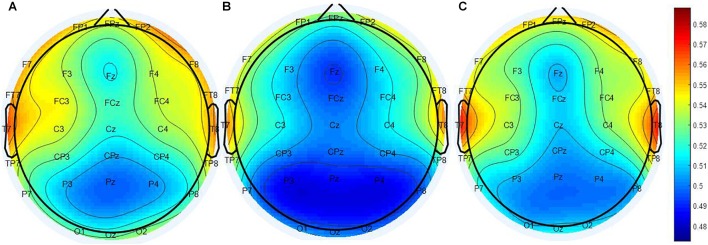
Scalp topographical maps of PEIMF2 index (resting state, eyes closed). From left to right: topographies of the **(A)** RP, **(B)** the NR, and **(C)** HC. PEIMF2 index in RP and HC groups is higher than NR group especially at left frontal electrodes.

The calculated PEIMF2 indices for the RP, NR and HC groups are plotted onto scalp topographic maps in Figure 2 with scales to the right of the maps. Greater PEIMF2 index are observed in RP and HC groups compared to NR, especially at left frontal sites.

**FIGURE 3:**
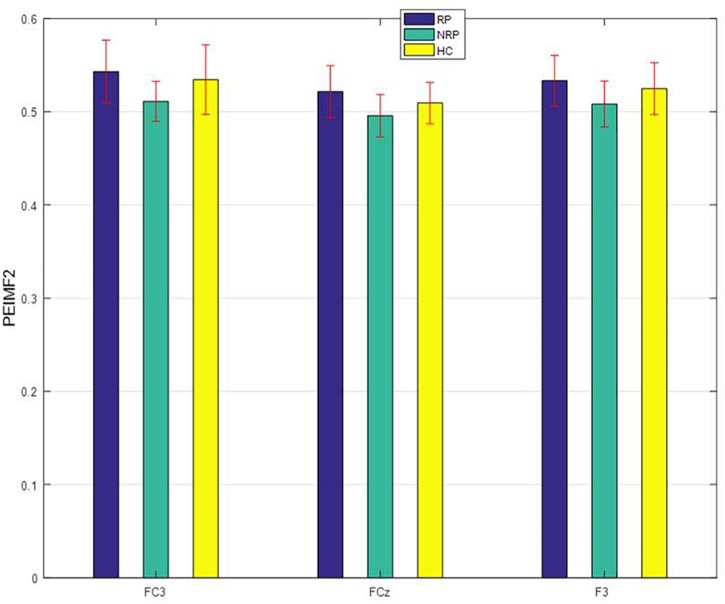
PEIMF2 index as a function of electrode sites for RP and NR with comparison to HC participant groups. Error bars represent ± 1 standard error. RP differ than NR to rTMS treatment especially at FC3.

One-way ANOVAs were calculated to investigate whether there were significant differences between RP and NR groups on PEIMF2 index for each electrode site (Table 2). The ANOVA showed that there were significant differences in the PEIMF2 index between the two groups in FC4 (*p* = 0.009), FCz (*p* = 0.001), F3 (*p* = 0.002), F4 (*p* = 0.003), Fz (*p* = 0.003), CP3 (*p* = 0.0049), P3 (*p* = 0.005) and FC3 (*p* < 0.001) electrodes. Also, as shown in Figure 3, the largest differences between RP and NR were observed at FC3 [RP = 0.543 (0.033), NR = 0.521 (0.028), HC = 0.533 (0.027) (mean (standard deviation))], FCz (RP = 0.511 (0.021), NR = 0.496 (0.029), HC = 0.508 (0.024)), and F3 (RP = 0.534 (0.037), NR = 0.501 (0.022), HC = 0.525 (0.028)) electrode sites. These results suggest that the PEIMF2 index may be able to differentiate between RP and NR groups, particularly at the frontal regions. There were no significant differences between RP and HC participants at these three electrode sites (*p*-value > 0.01), but there was a marginally significant difference between NR and HC (*p*-value << 0.01), where aHC exhibited a higher PEIMF2 index as compared to NR. Results were unchanged when taking in consideration treatment group as a covariate (i.e., HFL vs. iTBS stimulation).

**Table 2:** Results of the ANOVA investigating differences in PEIMF2 index between RP and NR groups at all electrode sites (α = 0.01).

Electrode site	*P-value*	Electrode site	*P-value*	Electrode site	*P-value*
FPz	0.107	FT7	0.108	TP8	0.024
FP2	0.123	FT8	0.481	Pz	0.275
FP1	0.120	Cz	0.022	**P3**	0.005
**Fz**	0.003	C3	0.01	P4	0.040
**F3**	0.002	C4	0.038	P7	0.014
**F4**	0.003	T7	0.524	P8	0.014
F7	0.053	T8	0.889	Oz	0.043
F8	0.054	CPz	0.251	O1	0.076
**FCz**	0.001	**CP3**	0.004	O2	0.012
**FC3^∗^**	<0.001	CP4	0.084		
**FC4**	0.009	TP7	0.566		

*FC3 asterisk denotes the most significant site for differentiating between RP and NR. Bold terms denote sites that significantly differentiate RP from NR*.

In this study, ROC curve analyses were used to explore the optimum component of EMD to undergo PE calculation in order to best differentiate between RP and NR patients. PE was computed for IMF1 to IMF4 to extract indices called PEIMF1 to PEIMF4 respectively, for FC3 electrode and multiple surrounding electrodes since this area best differentiated RP and NR. The AUC value for PEIMF2 index is 0.8, compared with 0.71 for PEIMF1, 0.76 for PEIMF3, and 0.74 for PEIMF4 in FC3 electrode and similar result gained from other electrodes. The AUC value of the ROC analysis classifying RP and NR was the greatest for PEIMF2 during resting state EEG, suggesting that PE calculated on the second IMF yields the best results for prediction of treatment response with moderate accuracy.

### Response Prediction Based on Frequency Band Measures

**FIGURE 4:**
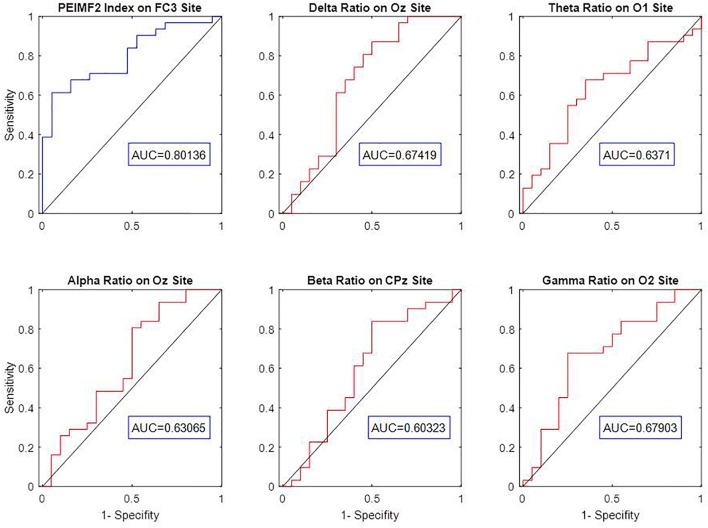
ROC curve analysis and AUC value of Delta, Theta, Alpha, Beta, Gamma relative powers to discriminate between RP an NR on best electrodes. The low AUC value of frequency band measures indicates weak prediction accuracy of these linear approaches.

Some conventional frequency band measures such as Delta, Theta, Alpha, Beta and Gamma relative powers were extracted from EEG signal of all electrodes. The efficiency of these indices was evaluated via AUC values on the best electrode for the classification of RP or NR. The AUC values of Delta at Oz, Theta at O1, Alpha at Oz, Beta at CPz and Gamma at O2 are 0.67, 0.64, 0.63, 0.60, and 0.68 respectively (Figure 4). The result show that there was a considerable difference between predictive value of PEIMF2 index (AUC = 0.8) and traditional frequency band measures on best electrode site.

## Discussion

The current study evaluated predictors of treatment response to rTMS administered to the left DLPFC in patients with TRD based on rsEEG signal. Our data shows that a non-linear entropy index, PEIMF2, yields superior outcome prediction performance compared to traditional frequency band indices. Our data indicate that TRD patients who responded to rTMS had higher entropy compared to NR, with the most prominent differences appearing in prefrontal areas (FCz, F3, and FC3 electrodes). Our findings extend previous the investigation of EEG-based biomarkers in depression, and position entropy indices applied in band-limited EEG components extracted with EMD method as a potential predictor for clinical use.

Entropy is becoming a valuable tool for the analysis of EEG activity and has received much attention in recent years in the study of brain disorders (Mizuno et al., 2010). Our data consistently show that NR patients have significantly lower entropy values in the prefrontal areas, and particularly the DLPFC region, compared to RP and HC subjects (Figure 3). Entropy indicates the complexity in a system, (Erguzel et al., 2015) and is also associated with the amount of “information” the signal carries. In the nervous system, higher levels of entropy have been consistently associated with healthy states where the nervous system is able to respond and adapt to dynamic changes. Conversely, lower entropy values (more regular, less information) are associated with pathological states and loss of the prime ability of the nervous system to respond to changes (Kevric and Subasi, 2017). A plausible hypothesis to explain the absence of difference between RP and HC would be that RP still have a system that is capable of such changes and this would make these patients amenable to respond to rTMS. Lower entropy levels in NR participants may indicate a reduction in typical intra-cortical information flow, a more regular, less complex EEG in the left frontal region and fewer chances for the system to change in response to rTMS. Therefore, lower levels of entropy (i.e., more regularity) may reflect a less preserved brain function that is not amenable to the effect of rTMS. This raises the question as to whether these patients would be amenable to a different type of stimulation (e.g., inhibitory rTMS), a different anatomical target (e.g., dorsomedial prefrontal cortex), or a higher dose of excitatory rTMS (e.g., more pulses per session or per day, accelerated protocols). Furthermore, our findings are convergent with those recently reported by Jaworska et al. (2018) who found that increased diffuse multi-scale entropy was predictive of treatment response to antidepressants and Farzan et al. (2017b) who showed that non-linear complexity measures was superior to power in explaining the therapeutic efficacy of seizure therapy.

The electrode sites that significantly distinguish RP from NR are located in left frontal (F3 and FC3), right frontal (F4, FC4), left parietal (CP3, P3) and central (FCz, Fz) sites (Table 2). A plausible explanation of our results is that the antidepressant effects of rTMS to the DLPFC are not restricted only to the local effect on the L-DLPFC, but rather that target circuits that underlie complex brain functions (e.g., affect regulation) including the frontal gyrus, anterior cingulate cortex, amygdala, and insula (Sheline et al., 2010; Smart et al., 2015). The current result converge with previous findings of brain areas related to MDD (Silverstein et al., 2015; Milev et al., 2016; Ge et al., 2017) and further extends it by adding new evidence that an entropy index indicating the complexity in a network could potentially serve as a predictor of clinical response to rTMS.

There are conflicting reports regarding the predictive capacity of frequency band metrics in the EEG such as alpha and theta activity. Some studies showed there was no correlation in alpha activity and treatment response (Price et al., 2008; Widge et al., 2013) whereas another showed that there was a negative correlation between the two (Micoulaud-Franchi et al., 2012). Moreover, one group reported that increased slow theta activity in the subgenual zone of the anterior cingulate cortex was correlated with positive response to rTMS (Hallett, 2007) while another group reported that theta rhythm increase in the frontal cortex is associated with non-response to rTMS treatment (Silverstein et al., 2015). Our own data would be convergent with the idea of a moderate predictive ability of frequency band (linear) metrics (Figure 4). One possible reason for the inferior predictive ability of linear metrics may be related to the non-linear nature of neural processes, as threshold and saturation phenomena control the dynamical behavior of individual neurons (Narushima et al., 2010). In contrast, our data supports the utility of non-linear metrics in predicting treatment outcome. Our measure quantified higher order non-linear complexity, which is not obtained using traditional EEG spectral-band analyses such as alpha or theta band power.

PEIMF2 measure is based on non-linear dynamics and has been found to indirectly index neuroplasticity (Hayley et al., 2005). PEIMF2 may represent the excitatory and inhibitory balance of the related networks in MDD which would also be associated with neuroplasticity. The scalp topography for the PEIMF2 values show that the most prominent differences between RP and NR groups are observed in the left frontal electrodes (Figure 3), which is consistent with previous findings of brain areas related to MDD. Considering this information, we would speculate that a plausible mechanism mediating the response to rTMS may be neuroplastic changes on relevant circuits involved in affect regulation and other symptomatic domains.

EMD adaptively and locally decomposes non-stationary EEG signals into a sum of IMFs that represent amplitude- and frequency- modulated components specific to the energy levels of individual patients (Shalbaf et al., 2012). EMD has shown better properties over other methods of EEG decomposition such as the short-time Fourier transform (STFT), independent component analysis (ICA) (Makeig et al., 1996) and wavelet transform (WT) (Zikov et al., 2006). For instance, the STFT excludes EEG features with a short duration or narrow frequency band. ICA is hampered by the intrinsically non-stationary nature and the non-linear couplings involved in neural signal generation. Moreover, WT forces the decomposition of the signal into a pre-defined set of basic functions, therefore temporal patterns of EEG signals cannot be obtained precisely. Conversely, EMD is a decomposition technique that is completely data-driven and thus utilizes empirical knowledge of oscillations intrinsic to the given time series (Baskaran et al., 2012). Furthermore, unlike wavelet analysis, EMD does not depend on a fixed set of basic functions; instead it searches for IMF embedded within the data. Therefore, these pitfalls of the other methods, or the strength of the data-drive EMD method may make it a better biomarker of treatment response.

Some limitations of our work should be considered in order to better interpret our results. First, the decomposition procedure of EMD requires the arbitrary choice of the stopping criteria for the sifting and the spline-fitting scheme (Sweeney-Reed and Nasuto, 2007). The former could lead to uniform or deviated IMFs, and the latter may result in problems overshooting or distorting the beginning and ending of signals. Second, we believe that a multi-modal approach of response prediction that holistically integrates a variety of sources of data including clinical, neuroimaging, and neurophysiological measures may be most reliable because many clinical factors may affect the central nervous system including baseline physiological and neurological differences, thus decreasing the predictive power of related EEG measures. Finally, we acknowledge this work provides a preliminary proof-of-principle evidence and the real consistency will only be determined with future larger trials or replication of the two methods of analysis.

To conclude, this study addresses a new method to decompose the neuronal oscillations with EMD to obtain a series of IMFs. The results show that the permutation entropy measure applied to the second IMF yields the optimal result in predicting treatment response. This seems to indicate that second IMF oscillation plays an important role in discriminating between RP and NR in the context of rTMS treatment. The method described herein certainly merits further research in larger samples to replicate and improve its predictive power. Prospective studies applying our predictor to decide treatment interventions may be used in the future, perhaps facilitating the precise prescription of rTMS as a first-line treatment when several favorable neurophysiological predictive factors are present, thus avoiding unsuccessful pharmacotherapy trials and expediting recovery.

## Ethics Statement

This study was carried out in accordance with the recommendations and approvals of UBC Clinical Research Ethics Board as well as the Vancouver Coastal Health Research Institute with written informed consent from all subjects in accordance with the Declaration of Helsinki. Trials were registered in ClinicalTrials.Gov, identifier NCT02800226 and NCT01887782.

## Author Contributions

RS, CB, and FV-R conceived and designed the study. DB, ZD, and RL provided input on the study design. RS, CB, and FV-R developed the plan for statistical analyses. RS analyzed the data. All authors contributed to the interpretation of data. RS and FV-R, drafted the manuscript. All authors made revisions to the manuscript.

## Conflict of Interest Statement

DB reports research grants from the Canadian Institutes of Health Research (CIHR), US National Institutes of Health, Weston Brain Institute, Brain Canada, the Temerty Family Foundation (through the Centre for Addiction and Mental Health Foundation and the Campbell Research Institute), and Brainsway; reports receiving in-kind equipment support for investigator-initiated studies (including this study) MagVenture; is the site principal investigator for three sponsor-initiated studies for Brainsway; and has been on an advisory board for Janssen Pharmaceutical. ZD reports research grants and equipment in-kind support for an investigator-initiated study from Brainsway and Magventure. JD reports research grants from CIHR, the National Institute for Mental Health, Brain Canada, the Canadian Biomarker Integration Network in Depression, the Ontario Brain Institute, the Klarman Family Foundation, the Arrell Family Foundation, and the Edgestone Foundation; reports travel stipends from Lundbeck and ANT Neuro; reports in-kind equipment support for this investigator-initiated trial from MagVenture; and is an advisor for and is an advisor for BrainCheck. RL reports research grants or consulting or speaking honoraria from Akili Interactive, Asia-Pacific Economic Cooperation, Allergan, AstraZeneca, Bristol-Myers Squibb, Canadian Depression Research and Intervention Network, Canadian Network for Mood and Anxiety Treatments, Johnson and Johnson, Lundbeck, Lundbeck Institute, MagVenture, Pfizer, St Jude Medical, Otsuka, and Takeda. FV-R reports research grants from CIHR, Brain Canada, Michael Smith Foundation for Health Research, and Vancouver Coastal Health Research Institute; reports receiving in-kind equipment support for this investigator-initiated trial from MagVenture; and has been on an advisory board for Janssen. CB, FF, RS, JT, and CP declare no competing interests.

## Abbreviations

DLPFC: dorsolateral prefrontal cortex
EMD: empirical mode decomposition
HDRS: hamilton depression rating scale
MDD: major depressive disorder
ROC: receiver operating characteristic
rTMS: repetitive transcranial magnetic stimulation
TRD: treatment-resistant depression.
