# Supplementary material for: Non-linear Entropy Analysis in EEG to Predict Treatment Response to Repetitive Transcranial Magnetic Stimulation in Depression
Source: Front Pharmacol. 2018 Oct 30;9:1188. doi: 10.3389/fphar.2018.01188 (PMC6218964; doi:10.3389/fphar.2018.01188)
Supplement: Supplementary file 1 [file Table_1.docx]

**Supplemental material**

**Inclusion/Exclusion Criteria for Treatment-Resistant Depression (TRD) Patients**

***Inclusion Criteria:***

Patients were included if they:

(1) were outpatients

(2) were voluntary and competent to consent to treatment

(3) had a Mini-International Neuropsychiatric Interview (MINI) confirmed diagnosis of MDD, single or recurrent

(4) were between the ages of 18 and 65 years

(5) had failed to achieve a clinical response to an adequate dose of an antidepressant based on an Antidepressant Treatment History Form (ATHF) score of > 3 in the current episode OR had been unable to tolerate at least 2 separate trials of antidepressants of inadequate dose and duration (ATHF 1 or 2)

(6) had a score > 18 on the HDRS-17 item

(7) had no increase or initiation of any psychotropic medication in the 4 weeks prior to screening

(8) were able to adhere to the treatment schedule

(9) passed the TMS adult safety screening (TASS) questionnaire

***Exclusion Criteria:***

Patients were excluded if they:

(1) had a history of substance dependence or abuse within the last 3 months

(2) had a concomitant major unstable medical illness, cardiac pacemaker or implanted medication pump

(3) had active suicidal intent at the time of screening

(4) were pregnant

(5) had a lifetime Mini-International Neuropsychiatric Interview (MINI) diagnosis of bipolar I or II disorder, schizophrenia, schizoaffective disorder, schizophreniform disorder, delusional disorder, or current psychotic symptoms

(6) had a MINI diagnosis of obsessive compulsive disorder, post-traumatic stress disorder (current or within the last year), anxiety disorder (generalized anxiety disorder, social anxiety disorder, panic disorder), or dysthymia, assessed by a study investigator to be primary and causing greater impairment than MDD

(7) had a diagnosis of any personality disorder, and assessed by a study investigator to be primary and causing greater impairment than MDD

(8) failed a course of electro-convulsive therapy (ECT) in the current episode or previous episode

(9) received rTMS for any previous indication due to the potential compromise of expectancy effects

(10) had any significant neurological disorder or insult including, but not limited to: any condition likely to be associated with increased intracranial pressure, space occupying brain lesion, any history of seizure except those therapeutically induced by ECT, cerebral aneurysm, Parkinson’s disease, Huntington’s chorea, multiple sclerosis, significant head trauma with loss of consciousness for greater than or equal to 5 minutes

(11) have an intracranial implant (e.g., aneurysm clips, shunts, stimulators, cochlear implants, or electrodes) or any other metal object within or near the head, excluding the mouth, that cannot be safely removed

(12) If participating in psychotherapy, must have been in stable treatment for at least 3 months prior to entry into the study, with no anticipation of change in the frequency of therapeutic sessions, or the therapeutic focus over the duration of the study

(13) had a clinically significant laboratory abnormality, in the opinion of the one of the principal investigators

(14) were currently (or in the last 4 weeks) taking lorazepam greater than 2 mg daily (or equivalent) or any dose of an anticonvulsant, due to the potential to limit rTMS efficacy

(15) had a non-correctable clinically significant sensory impairment (i.e., cannot hear well enough to cooperate with interview).

(16) had failed more than 3 adequate trials (ATHF > 3) of medication in the current episode.

**Inclusion/Exclusion for Healthy Comparison (HC) Subjects**

***Inclusion Criteria:***

Participants were included if they:

(1) were voluntary and competent to consent to the study

(2) were between the ages of 18 and 65

(3) were fluent in English, sufficient to complete interviews and cognitive testing

(4) had no history of Axis I or Axis II disorders, as determined by the Mini-International Neuropsychiatric Interview (MINI)

***Exclusion Criteria:***

Participants will be excluded if they:

(1) had a lifetime MINI diagnosis of bipolar I or II disorder, schizophrenia, schizoaffective disorder, schizophreniform disorder, delusional disorder, or current psychotic symptoms

(2) had a MINI diagnosis of obsessive compulsive disorder, post-traumatic stress disorder (current or within the last year), anxiety disorder (generalized anxiety disorder, social anxiety disorder, panic disorder), dysthymia or any personality disorder

(3) had history of mood disorders or psychosis in first degree relative (parents, siblings, offspring)

(4) were unable to provide family history of biological family (i.e., adopted persons were not eligible)

(5) had a history of substance dependence within the last 3 months

(6) had a concomitant major unstable medical illness

(7) had any significant neurological disorder or insult including, but not limited to: any condition likely to be associated with increased intracranial pressure, space occupying brain lesion, cerebral aneurysm, Parkinson’s disease, Huntington’s chorea, multiple sclerosis, significant head trauma with loss of consciousness for greater than or equal to 5 minutes

(8) had a non-correctable clinically significant sensory impairment (i.e., cannot hear well enough to cooperate with interview).

(9) had a personal or family history of seizures
